# Gastrointestinal symptoms of infantile colic and their change after light needling of acupuncture: a case series study of 913 infants

**DOI:** 10.1186/1749-8546-6-28

**Published:** 2011-08-11

**Authors:** Marianne Reinthal, Iréne Lund, Dacil Ullman, Thomas Lundeberg

**Affiliations:** 1 Department of Paediatrics, Sahlgrenska Academy, Göteborg University, SE-405 30 Göteborg, Sweden; 2 Mariannes Akupunktur, C W Borgs väg 3, SE-444 31 Stenungsund, Sweden; 3 Department of Physiology and Pharmacology, Karolinska Institutet, SE-171 77 Stockholm, Sweden; 4 Barrskogsgatan 11, SE-412 74 Göteborg, Sweden; 5 Foundation for Acupuncture and Alternative Biological Treatment Methods, Sabbatsbergs Hospital, SE-102 34 Stockholm, Sweden

## Abstract

**Background:**

Infantile colic is a common painful clinical condition associated with signs of distended intestines and an increase in colon peristalsis. However, clinical documentation of observed gastrointestinal functions in the condition is still lacking. Even though the ailment is common, no clear treatment guidelines exist. While acupuncture with minimal stimulation has been shown to be effective in reducing crying behaviour of infants suffering from colic, the documented effect of acupuncture on gastrointestinal function in children with infantile colic is scarce. This case series study aims to document the symptoms of routinely rated gastrointestinal function and the changes in these symptoms after minimal acupuncture in a larger group of children with infantile colic.

**Methods:**

This study included 913 infants with normal weights, and lengths at birth. The infants' mean age was 5.4 weeks when the observations started, and had colic symptoms since two weeks after birth. Light needling stimulation of the acupuncture point LI4 was performed for 10-20 seconds bilaterally on a daily basis for a mean of 6.2 consecutive days. A questionnaire with verbal rating scales for the parents' evaluation was used before and after the treatment period.

**Results:**

Before treatment the infants were assessed by the parents in terms of 'often have inflated stomachs' (99%) and 'seldom drool' (76%), 'regurgitate' (53%) and 'belch' (62%). Moreover, the reported frequency of defecation was 5-8 times per day (64%), with a yellowish-green colour (61%) and with a water-thin consistency (74%). After treatment, the variables of inflated stomachs, drooling and regurgitating were systematically changed, and rated by the parents as occurring 'sometimes' while belching was rated as occurring 'often' and the frequency of defecation was reduced to 1-4 times/day with a mustard yellow colour and a gruel-like consistency. The parents also rated their impression of the infants' general colic symptoms including crying behaviour as much ameliorated in 76% of the cases.

**Conclusion:**

The results of the present study show that minimal acupuncture at LI4 in infantile colic is an effective and easy treatment procedure that, furthermore, is reported to be without serious side effects.

## Background

Infantile colic is reported to have an incidence rate between 5% and 19% in prospective studies on infants aged less than three months [1,2]. The classical definition of infantile colic is 'a seriously fussy or colicky infant who is otherwise healthy and well fed but has paroxysms of irritability and fussing or crying, more than three hours per day, more than three days per week for more than three weeks, or symptoms so severe that medication is indicated' [3], and this definition is still valid for diagnosis [1]. Thus, the clinical diagnosis is based on the children's crying behaviour characterized by paroxysmal and inconsolable crying predominantly in the early night hours and a body language with flexed knees, clenched fists and a grimacing face, often flushed, together expressing a painful state, despite the fact that crying is an unspecified and multifactorial communication of infants, reflecting different reasons for their dissatisfaction including pain [4].

Pathogenesis of infantile colic is unclear but may be related to food allergy, flatulence, intestinal hormonal imbalances, parental factors and dysregulation of the autonomic nervous system [1,2,5]. Infantile colic is a painful condition associated with extensive gas production in distended intestines [6,7] and increased colon peristalsis [8].

The characteristic intense crying of infantile colic can be a risk for the trigging of the shaken baby syndrome [9]. However, there are still no clear guidelines for treatment [10]. Both pharmacological and non-pharmacological treatments have been tested leading to varied effects including undesirable side effects [11]. A common pharmacological treatment is Simethicone (Minifom ^®^) with the purpose of reducing surface tension of gas in the intestines thereby reducing the pain; however, a controlled trial concluded that the Simethicone treatment was not superior to placebo [12]. Dicyclomine, an anti-cholinergic drug with spasmolytic effects, has been tested and serious side effects were reported, including drowsiness, constipation, loss of motion and apnoea [13]. Dicyclomine is now contraindicated in infants younger than six months [14]. Dicyclomine is no longer a therapeutic option [15].

Among the non-pharmacological treatment regimes, acupuncture with minimal stimulation (*ie *light needling) [16,17] has been demonstrated to be effective in treating crying symptoms of infantile colic [18,19]. The parents rated the needling as more effective for decreasing these symptoms than care without needling. Some parents also reported that the pattern of belching and flatulence (having a gas-extended stomach) accompanied by crying was changed after minimal acupuncture. This finding is interesting as a growing clinical experience suggests that there may be disturbed gastrointestinal function among children with infantile colic. Previous studies have, however, considered the baby's crying behaviour or influence on the parental interaction with their babies as the primary outcome. These studies were small in size and have not systematically evaluated the rated symptoms of affected gastrointestinal function, or the use of acupuncture in routine care. Treating the condition with acupuncture is based on the expected physiological changes in gastrointestinal function induced by the needle stimulation and the following response in afferent nerve activity. During the insertion of the needle, the sympathetic tone is increased, generating a decreased gastrointestinal activity. After acupuncture, the autonomic activity may be characterized by an increased parasympathetic tone as well as a decreased sympathetic tone resulting in increased gastrointestinal motility [20-22].

The present study aims to demonstrate the symptoms of routinely rated gastrointestinal function in children with infantile colic and the rated changes in these symptoms after treatment with light needling (acupuncture). The present study is, for ethical reasons, not designed for the evaluation of the treatment efficacy by testing the hypothesis of no change related to a control group, but as an extended case series study.

## Methods

### Study design

The study was approved by the Human Ethics Committee at Göteborg University, (M2) 14/8 2008, Dnr: 430-08 to conduct a retrospective case series study in normal clinical practice with the parents' ratings of gastrointestinal symptoms of infantile colic before and after treatment with light needling. The data were collected consecutively during a fixed time period between January 2003 and December 2007. Thus, the sample size *per se *was not determined before the study.

### Subjects and clinical settings

The infants participating in this study, aged 0-12 weeks, were recruited when their parents consulted the acupuncture clinic for colic treatment. All infants were healthy with normal bodyweight and length according to the medical examination immediately after birth. All children were registered at the local Child Welfare Clinics for regular check-ups supervised by registered nurses specialised in children's welfare. All infants included in this study were diagnosed to have infantile colic according to the aforementioned definition, with paroxysms of inconsolable crying for more than three hours a day and more than three days per week and with a body language of pain generated from the gastrointestinal tract.

If the acupuncture treatment was deemed appropriate for the infant after clinical assessment, the parents were asked to complete a standardised questionnaire before and after the treatment. The acupuncture was performed by a registered nurse and midwife practicing acupuncture on children with infantile colic for 12 years in a clinic run in close co-operation with Child Welfare Clinics within an area of western Sweden.

### Questionnaire

A questionnaire tailored to assess gastrointestinal symptoms was sought before the start of data collection for the study. As nothing suitable was found, a questionnaire was constructed based on the clinical experience of MR.

At the first visit, prior to treatment, the parents answered questions on the child's medical history (Table 1). The parent's ratings of their child's current gastrointestinal symptoms were recorded in the questionnaire with seven verbal rating scales consisting of three to four response categories each (Table 2). The parents were asked to complete the same questionnaire after the treatment and it was, therefore, given to them at the penultimate treatment in order for them to return with it at the final treatment session. Moreover, the parents were asked to use a five-category verbal scale ('much worse, slightly worse, no change, slightly ameliorated, much ameliorated') to rate their opinions about changes of their child's general colic symptoms. Before the acupuncture sessions, all the children were breastfed or fed with formula free of cow's milk protein. All questionnaires were encoded with names and participant numbers when the data were entered into data sheets.

**Table 1:** Data of medical history (*n *= 913)

Variable	Mean (SD)/N (%)
**Gestational age, weeks**	39.1 (1.8)
**Start of colic symptoms, age in weeks**	1.6 (1.2)
**Bodyweight, kg**	
*at birth*	3.45 (0.55)
*at start of the study*	4.58 (0.77)
**Length, cm**	
*at birth*	50.1 (2.4)
*at start of the study*	55.4 (2.8)
**Prevalence of colic symptoms as infant**	
*Mother*	298 (33%)
Yes	515 (57%)
No	93 (10%)
Don't know	235 (26%)
*Father*	501 (55%)
Yes	168 (19%)
No	
Don't know	
**Colic symptoms in 593 biological siblings**	348 (59%)

**Table 2:** Rated variables before and after light needling treatment

Variables	Response options
1. Regurgitation	Seldom, Sometimes, Often
2. Belching	Seldom, Sometimes, Often
3. Drooling	Seldom, Sometimes, Often
4. Being inflated in the stomach	Seldom, Sometimes, Often
5. Frequency of defecation	> 8 times/day, 5-8 times/day, 1-4 times/day, < 1 time/day
6. Faecal colour	Green, Yellowish-green, Mustard yellow, Light yellow
7. Faecal consistency	Water-thin like, Mucous like, Gruel like, Tooth paste like

### Treatment

The acupuncture treatment consisted of light needling stimulation of the acupuncture point LI4 located in the first dorsal interosseus muscle of the hand. A thin, short (0.20 × 15 mm), sterile and disposable acupuncture needle was inserted 1-3 mm in the infant's hand, lightly manipulated for a few seconds until a certain sensation of resistance was perceived in the needle, and then left in place for approximately10-20 seconds before withdrawal. Apart from this, no other specific response was sought during the treatment and no infant expressed or had an overt reaction to the needle insertion. The same procedure was repeated on the infant's other hand. The treatment was performed once daily for approximately one week (*ie *5-8 sessions total). This superficial and short-term stimulation was chosen because it was demonstrated to be effective in previous studies [18,19]. The infants' mothers were advised to avoid cow's milk protein [23].

### Statistical analysis

The data of the medical history were presented as mean and standard deviation (SD). The discrete data of rated subjective variables were presented as medians and frequencies. The distribution of the response frequencies was shown in histograms and contingency tables where the cells in the grey-shaded main diagonal demonstrated no change in rating.

The hypothesis of no change in the paired assessments of ratings within the group before as compared with after treatment was analyzed with the Sign test with correction for continuity. In addition, the changes in the paired data in different variables were further analysed by a rank-based, non-parametric method formulated by Svensson in order to estimate the size of the systematic, group-related changes as well as varied results related to the individuals [24,25]. A systematic change in assessments before compared with after light needling appeared as different marginal frequency distributions and defined the measure of relative position (RP) with possible values ranging from -1 to 1, where RP = 0 means a lack of change between the two assessments. The presence of an individual variation in change, not explained by the systematic change related to the group, in this case demonstrating dispersed responses among the parents, was calculated as the relative rank variance (RV) ranging from 0 to 1. The RP and RV values were presented with their 95% confidence interval (CI) and values were considered significant when the confidence interval did not cover 0. The extent of dispersed responses was evident from the contingency tables. The software package of Statistica 9.0 (StatsSoft^® ^Scandinavia AB, Uppsala, Sweden) was used for descriptive statistics and statistical analysis with Sign test. A two-side *P *value less than 0.001 was regarded as significant for test of no change in rated variables before as compared with after treatment where the individual *P *values were adjusted for multiple tests according to Holm [26]. For conduct of the rank-invariant method by Svensson, the software package of Sysran 1.0 SYSRAN V.1.0 (JK Biostatistics, Sweden) for Matlab V.6.0 (The MathWorks, USA) was used.

## Results

The data of 987 treated infants with colic were collected consecutively in a series. The data from 74 infants were excluded due to incomplete questionnaires of rated baseline data, though no parents refused to complete them. The collected data from observations of a total of 913 infants (girls, *n *= 409; boys, *n *= 504) aged 5.4 (SD 2.5) weeks, with normal weight and length at birth but with colic symptoms since the second week (mean value) of life were included, (Table 1). Approximately 30% of the infants' parents reported that they themselves had suffered from colic symptoms as their children did. Furthermore, 59% of the biological siblings of the affected children in the present study also had symptoms when they were infants.

### Symptoms of intestinal function before treatment

**Figure 1:**
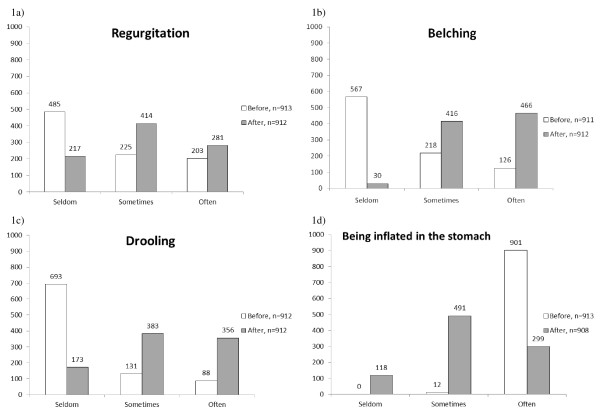
**Frequency histograms of rated varied gastrointestinal symptoms**.

**Figure 2:**
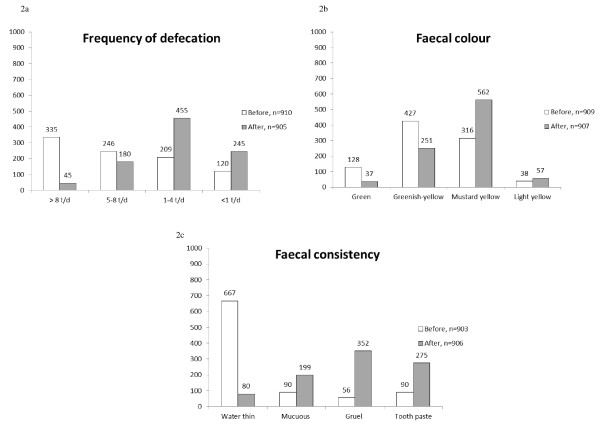
**Frequency histograms of rated gastrointestinal (feacal) symptoms**.

The observed frequency of regurgitation was rated median *seldom *(range: seldom to often) in 485 (53%) of the 913 infants (Figure 1a), and also belching was rated median *seldom *(range: seldom to often) in 567 (62%) of the 912 children (Figure 1b). Drooling frequency was rated to be median *seldom *(range: seldom to often) in 693 (76%) of the 912 infants, *ie *perceived by the parents as their child having a 'dry mouth' when they tried to give the child a pacifier (Figure 1c) and the most frequently reported symptom before treatment was inflated stomach, reported median as *often *(range: sometimes to often) in 901 of the 913 (99%) infants (Figure 1d). Furthermore, symptoms more strictly related to the intestinal function such as frequency of defecation, faecal colour and faecal consistency were rated. Before treatment the rated frequency of defecation was median *5-8 times per day *in 581 (64%) of the 910 infants, and with a median rated *yellowish-green *faecal colour in 555 (61%) of the 909 infants. The faecal consistency was rated as median *water-thin *in 667 (74%) of the 903 infants (Figure 2a-c).

### Changes in intestinal function after treatment

The infants were given 6.2 (SD 1.1) acupuncture sessions. The post-treatment changes as rated by the parents are reported as follows.

### Regurgitation

**Figure 3:**
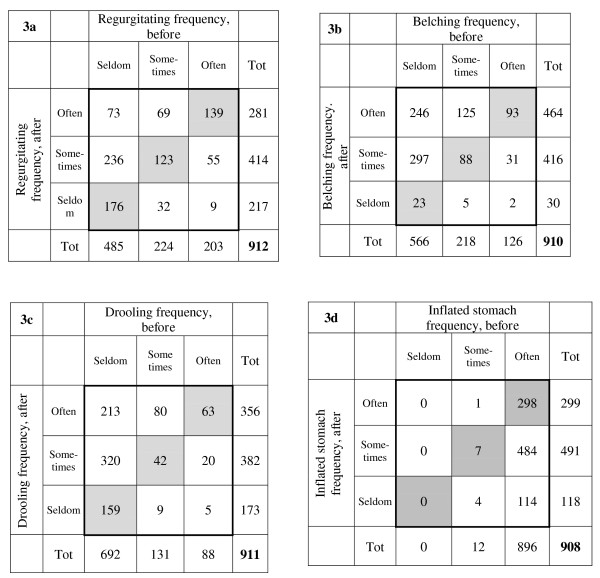
**Paired data of rated regurgitation (a), belching (b), drooling (c), and being inflated in the stomach (d) before and after light needling treatment**. Tot = Total marginal frequency in respective category.

The observed frequency of regurgitation changed to median *sometimes *(range: seldom to often) after treatment (Figure 1a). According to the paired data shown in Figure 3a, the frequency of regurgitation was rated to be the same in 438 (48%) of the 912 infants, increased in 378 (41%) and decreased in 96 infants (11%) after treatment compared to before treatment, p < 0.001. The marginal frequency distribution (the group-related effect) indicated a shift towards more frequent regurgitation after treatment compared to before treatment (measured as relative position, RP 0.27; 95%CI 0.23 to 0.31). The individual ratings, measured as the relative rank variance (RV) were consistent among the parents (RV 0.09; 95%CI 0.07 to 0.11).

### Belching

The belching frequency was also reported as changed after treatment, now being median *often *(range: seldom to often), (Figure 1b). In 204 (23%) of the 910 infants, the belching frequency was unchanged whereas it was rated more frequent in 668 (73%) and less frequent in 38 (4%) of the infants, p < 0.001. The shift for the whole group to a higher frequency was evident (RP 0.65; 95%CI 0.61 to 0.68) although the ratings were slightly dispersed at the parents' individual level (RV 0.13; 95%CI 0.10 to 0.16) (Figure 3b).

### Drooling

After treatment, the infants' drooling behaviour changed to median *sometimes *according to the parents' rating, now being median *sometimes *(range: seldom to often) (Figure 1c). In 264 (29%) infants drooling was unchanged whereas in 613 (67%) it increased, and in 34 (4%) it was reported as decreased, p < 0.001, *ie *a systematic shift towards increased salivation appeared, (RP 0.59; 95%CI 0.55 to 0.62), with negligible individual variations in the opinion (RV 0.08, 95%CI 0.05 to 0.10) (Figure 3c).

### Inflated stomach

The parents' rating of inflated stomach was markedly changed to median *sometimes *(range: seldom to often) after treatment (Figure 1d). Detailed information from the paired data showed that the symptom was rated unchanged in 305 of 908 (34%) infants, increased in one infant and decreased in 602 (66%), p < 0.001. This response pattern was confirmed by the systematic change towards less frequent (RP -0.66; 95%CI -0.69 to -0.63), with negligible individually dispersed values (RV 0.004, 95%CI 0.00 to 0.01) (Figure 3d).

### Frequency of defecation

**Figure 4:**
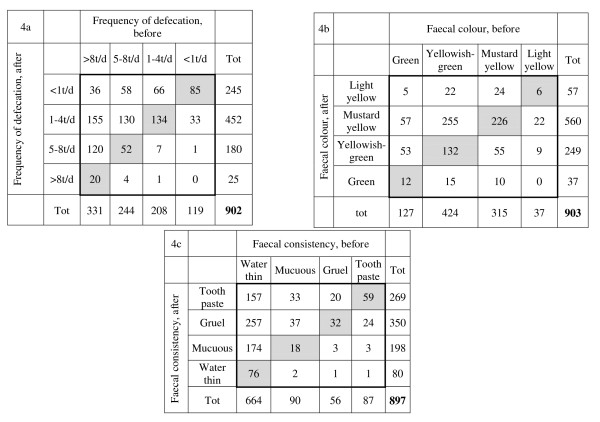
**Paired data of rated frequency of defecation (a), faecal colour (b), and faecal consistency (c) before and after light needling treatment**. Tot = Total marginal frequency in respective category; t/d = times/day.

The frequency of defecation was median *1-4 times/day *(range: < 1 time/day to > 8 times/day) after treatment (Figure 2a) and systematically changed in position towards a decreased frequency since the paired data showed unchanged frequency in 291 (32%), increased in 46 (5%) and decreased in 565 (63%) of the 902 infants, p < 0.001, (Figure 4a) (RP -0.47; 95%CI -0.51 to 0.-44). The individual ratings were slightly dispersed (RV 0.12; 95%CI 0.09 to 0.13).

### Faecal colour

The colour of the faeces was rated as median *mustard yellow *(range: green to light yellow) after treatment (Figure 2b). In 416 infants (46%) the colour changed to more yellowish while the colour was unchanged in 376 (42%) and changed to a greenish colour in 111 (12%) of the 903 infants, p < 0.001, (Figure 4b). Overall a systematic change towards a yellowish colour was clear (RP 0.31; 95%CI 0.27 to 0.35), with a slight individual rating among the parents (RV 0.18; 95%CI 0.14 to 0.21).

### Faecal consistency

After treatment, the faecal consistency was rated as changed to median *gruel-like *(range: water-thin like to toothpaste-like) (Figure 2c). Paired data showed that in 185 (21%) of the 897 children the rated faecal consistency was unchanged, changed towards thinner consistency in 34 (4%) and towards more solid consistency in 678 (75%) infants, p < 0.001 (Figure 4c), which also was indicated by the measure for relative position (RP 0.67; 95%CI 0.63 to 0.70) with a slight variation at the individual level (RV 0.10; 95%CI 0.07 to 0.13).

### Overall impression of changed colic symptoms

**Figure 5:**
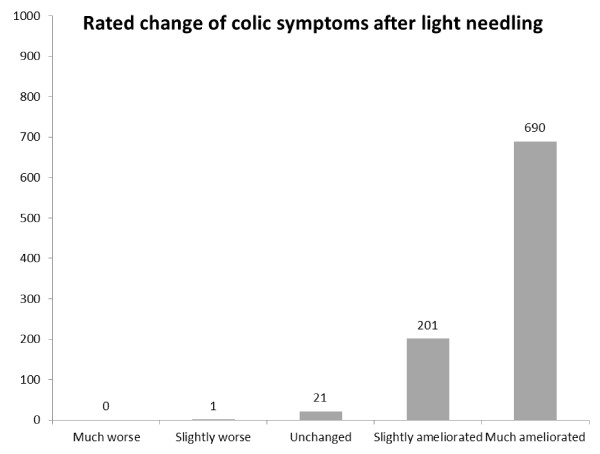
**Rated effect of treatment on generally colic symtoms, n = 913**.

The parents rated their perceived impressions of their children's general changes of colic symptoms (including crying behaviour) as much ameliorated in 76%, slightly ameliorated in 22% and unchanged in 2% of the 913 infants. In only one case was the situation perceived as slightly worse (Figure 5).

## Discussion

The results of this study show that the rated characteristic symptoms of the babies with infantile colic were 'dry mouth' when they were given a pacifier, symptoms of often having an inflated stomach, and a frequency of defecation of 5-8 times a day with water-thin consistency and a yellowish-green colour. When analysing the parents' observations, we found it clear that most symptoms were significantly changed after the treatment period. Drooling was found to be increased and there were fewer reports of inflated stomachs, as well as a lower rate of defecation. Despite possible multifactorial causes for the condition among the many children, the parents' reports after treatment were overall systematic in the group with only some degree of individual variation. The infants' parents also rated their general impression of changed colic symptoms (including crying) after treatment period as systematic ameliorated.

Crying of colicky infants, and of normal infants, is common during the evenings. A total of about 50% of the crying occurs between 6 pm and midnight [18,27]. The cause of this diurnal rhythm is not known. In an acoustic analysis, the quality of crying in colicky infants was higher pitched and dysphonic than that of non-colicky ones [28], suggesting that this rating could be used as an important outcome assessment for the treatment. In this study, general symptoms of infantile colic (including crying) were significantly reduced after treatment. This finding cannot solely be explained by parents' expectations (*ie *placebo) even though only motivated parents sought consultation as minimal acupuncture has been reported to be superior to general care in two randomised controlled trials [18,19]. Another possible confounding factor is that this condition naturally tends to resolve, thereby possibly including 'false positive' reports. However, in the two previous controlled trials, the significant difference between the light needling and the control group indicated that the influence of the natural resolution would not solely explain the outcome.

Intense crying is not only distressing to the mother but to the whole family as well [9]. Several studies have reported an association between family tension and infantile colic [1]. It has also been suggested that infantile colic predisposes for recurrent abdominal pain, and for allergic and psychological disorders [2]. A safe intervention that reduces colicky behaviour in infants is important both to the infant and the family.

As mentioned above, characteristic of the 913 babies suffering from infantile colic in this study were symptoms of dry mouth, often being troubled by gas in the stomach, and a high defecation frequency. Possibly, this may be attributed to dysfunction in the autonomic modulation of the gastrointestinal motility and functioning. However, in a recent study on factors associated with defecation patterns in infants aged 0-24 months [29], the reported defecation rate in a subgroup of children with infantile colic was lower than that in our study while they found a high frequency of defecation in the main group of subjects in their study, possibly related to immaturity of the gut and breast feeding. Therefore, the drop in defecation frequency could point to a maturation of the water-conserving capacity of the gut.

Following minimal acupuncture, drooling was increased and possibly the intestines were to a lesser degree extended by gas, suggesting that as a result of the treatment the sympathetic tone had decreased and/or the vagal tone had increased.

The vagus nerve is an important component in the regulation of the autonomic nervous system [30] composed of afferent sensory nerves and efferent motor nerves that innervate most inner organs, including skin and muscle tissue of the hand. Low baseline vagal activity is associated with infant risk conditions such as prematurity and depression. For example, preterm infants exhibit lower baseline vagal activity than full term infants, and infants who exhibit lower levels of vagal activity also exhibit fewer optimal neural developmental outcomes [30]. Vagal stimulation may therefore promote growth and development in preterm infants and improve developmental outcomes such as weight gain and gastric motility [31]. Sensory stimulation in the form of massage applied with moderate pressure may result in increased activity of vagal afferent nerve fibres projecting to the vagal nucleus of the solitary tract and a modulation of autonomic efferent activity. This is supported by studies showing that moderate pressure massage results in decreased heart rate, lower blood pressure and reduced stress hormone levels. Acupuncture is another mode of sensory stimulation, based on activation of mechanoreceptors and subsequent afferent nerve activity. Acupuncture lasting for 20-30 minutes has been shown to decrease sympathetic tone and to increase parasympathetic tone [21,22]. This dual effect on the autonomic regulation [32] suggests that acupuncture, including minimal needling, may have a more profound effect as compared to massage on conditions characterized by autonomic dysregulation. As such, minimal acupuncture of LI4 may result in the activation of mechanoreceptors and an increased sympathetic tone during the needle insertion, followed by an increased parasympathetic tone and a decreased sympathetic tone, resulting in increased (synchronized) gastrointestinal functioning. This suggestion is supported by the behavioural changes seen following acupuncture in colicky infants. The hypothesis generated from the results of this study is that effects of acupuncture in infantile colic may be related to an influence of the nervous system and has to be tested in further experimental studies. The present case series study was performed in a common clinical practice and as such the observations of the studied infants are likely to represent what it is seen in a regular clinical practice However, being a case series study it has its limitations.

## Limitations of the study

The present study is an extended case series study of 913 children meaning that it by definition lacks a control group, thereby not allowing for general interpretations referring to the studied group except for general systematic observations, *ie *that some symptoms in these infants were rated as improved in association with acupuncture treatment. However, one symptom (regurgitation) was worse. As the parents were asked to rate various symptoms and its changes, the results were inevitably influenced by the parents' interpretations of the symptom scores. The outcome measurements were not fully validated although the questionnaire was carefully designed. Possibly, there is also a considerable risk of measurement bias, as the advice to reduce cow's milk protein may be a confounder. However, this could also have been a confounding factor in one of the previous mentioned controlled trials [18] since this food restriction was adopted by many of the participating children's mothers but, without influencing the difference in outcome between the acupuncture group and the control group. As the duration of baby colic is often shorter than three months, it is tempting to suggest that the most important treatment is treatment itself and the choice of treatment is of less importance. If an intervention can result in some weeks of relief, the natural ending of colic will be closer. The results reported by the parents suggest that acupuncture may be tried in infantile colic, especially since worsening of symptoms was reported for only one infant. However, other treatments including administration of massage, sucrose solution, herbal tea, or hydrolyzed formula may also be tried [33].

A multi-factorial condition such as infantile colic would probably benefit from multifactorial treatment regimes. It would be also interesting to see if there is a synergistic effect if two or more treatments were combined (acupuncture, massage, sucrose solution, herbal tea, or hydrolyzed formula).

Before administration of a treatment or a combination of treatments in baby colic, medical examination and dietary instructions should be provided as well as parental counseling and information about the nature of the condition.

Studies such as the present one, and the study by Arikan *and collaborators *(2008) [33], are important as they demonstrate safe and cost effective methods for relief of infantile colic and related symptoms, as well as methods that can be taught to and administered by parents or in most health care settings. In a condition like infantile colic, that is common worldwide, the benefits of acupuncture treatment are obvious: many infants will achieve symptom relief from a simple intervention that may be provided by many health care providers.

## Conclusion

The results of the present study show that minimal acupuncture at LI4 in infantile colic is an effective and easy treatment procedure that, furthermore, is reported to be without serious side effects.

## Abbreviations

LI: large intestine; RP: relative position; RV: relative rank variance; CI: confidence interval.

## Competing interests

MR works at the acupuncture clinic. All other authors declare that they have no competing interests.

## Authors' contributions

MR designed the questionnaire and performed the treatments. DU collected the data together with MR. TL conceived the idea of the study and possible mechanisms of the treatment. IL and DU analysed the data and reported the results. All authors contributed equally to the writing and finalising of the manuscript and read and approved the final version of the manuscript.
